# UGT1A1 polymorphism has a prognostic effect in patients with stage IB or II uterine cervical cancer and one or no metastatic pelvic nodes receiving irinotecan chemotherapy: a retrospective study

**DOI:** 10.1186/s12885-020-07225-1

**Published:** 2020-08-05

**Authors:** Hideki Matsuoka, Ryusuke Murakami, Kaoru Abiko, Ken Yamaguchi, Akihito Horie, Junzo Hamanishi, Tsukasa Baba, Masaki Mandai

**Affiliations:** 1 Department of Gynecology and Obstetrics, Kyoto University Graduate School of Medicine, 54 Kawahara-cho, Shogoin, Sakyo-ku, Kyoto, 606-8501 Japan; 2 Department of Obstetrics and Gynecology, Kyoto Katsura Hospital, 17 Yamadahirao-cho, Nishikyo-ku, Kyoto, 615-8157 Japan; 3 Department of Gynecology, Shiga General Hospital, 5-4-30, Moriyama, Moriyama-city, Shiga 524-8524 Japan; 4 Department of Obstetrics and Gynecology, National Hospital Organization Kyoto Medical Center, 1-1 Fukakusa Mukaihata-cho, Fushimi-ku, Kyoto, 612-8555 Japan; 5 Department of Obstetrics and Gynecology, Iwate Medical University School of Medicine, 2-1-1, Idaidori, Yahaba, Iwate 028-3695 Japan

**Keywords:** Irinotecan chemotherapy, Uterine cervical cancer, *UGT1A1* polymorphisms

## Abstract

**Background:**

Uridine diphosphate glucuronosyltransferase 1 family polypeptide A1 (*UGT1A1*) is a predictive biomarker for the side-effects of irinotecan chemotherapy, which reduces the volume of tumors harboring *UGT1A1* polymorphisms. We aimed to determine whether *UGT1A1* polymorphisms can predict progression-free survival in patients with local cervical cancer treated with irinotecan chemotherapy.

**Methods:**

We retrospectively analyzed the data of 51 patients with cervical cancer treated at a single institution between 2010 and 2015. All patients were diagnosed with 2009 International Federation of Gynecology and Obstetrics (FIGO) stage IB1, IB2, IIA, or IIB squamous cell carcinoma, underwent radical hysterectomy, and received irinotecan chemotherapy as neoadjuvant and/or adjuvant treatment. All patients were examined for irinotecan side effects using *UGT1A1* tests. Conditional inference tree and survival analyses were performed considering the FIGO stage, age, the *UGT1A1* status, and the number of metastatic lymph nodes to determine primary factors associated with progression-free survival.

**Results:**

The tree-structured survival model determined high recurrence-risk factors related to progression-free survival. The most relevant factor was ≥2 metastatic lymph nodes (*p* = 0.004). The second most relevant factor was *UGT1A1* genotype (*p* = 0.024). Among patients with ≤1 metastatic lymph node, those with *UGT1A1* polymorphisms benefited from irinotecan chemotherapy and demonstrated significantly longer progression-free survival (*p* = 0.020) than those with wild-type *UGT1A1*.

**Conclusions:**

Irinotecan chemotherapy might be beneficial in patients with cervical cancer, *UGT1A1* polymorphisms, and ≤ 1 metastatic lymph nodes.

## Background

In 2018, cervical cancer caused approximately 311,000 deaths worldwide and was the fourth leading cause of cancer-related deaths in women [1]. Among women younger than 40 years, it is the second most common cancer and the third deadliest [2]. In Japan, 2900 women die from cervical cancer every year, and the mortality of cervical cancer is increasing due to insufficient awareness of human papillomavirus (HPV) vaccination and low rates of cancer screening [3]. It is important to decrease the morbidity and mortality of cervical cancer. In the Japan Society of Obstetrics and Gynecology’s annual patient report for 2015, the 5-year survival rates of patients with 2009 International Federation of Gynecology and Obstetrics (FIGO) stage I, II, III, and IV cervical cancer were 92.1, 74.2, 52.0, and 29.8%, respectively [4].

The National Comprehensive Cancer Network guideline and the Japan Society of Gynecologic Oncology guidelines recommend concurrent chemoradiotherapy (CCRT) as adjuvant therapy for cervical cancer patients at a high risk of recurrence after surgery [5, 6]. However, in Japan, adjuvant chemotherapy for local cervical cancer following radical hysterectomy is performed in about 13% of cervical cancer patients because of the severe adverse effects of adjuvant radiotherapy (RT) [3, 4]. Jung et al. reported that stage IB-IIA cervical cancer could benefit from adjuvant chemotherapy after radical hysterectomy (RH), with fewer long-term complications and non-inferior therapeutic effects to adjuvant radiotherapy [7]. Matsuo et al. reported that postoperative systematic chemotherapy and CCRT have similar survival outcomes for clinical stage IB-IIB cervical cancer patients who are undergoing radical hysterectomy and are diagnosed with lymph node metastasis by histopathological findings. Chemotherapy is independently associated with lower rates of distant recurrence, but higher rates of local recurrence than CCRT [8]. Takekuma et al. reported that chemotherapy after surgery for high-risk patients had a similar efficacy but a different toxicity profile than that of CCRT, which is associated with worse toxicity than chemotherapy [9]. In Japan, phase II trials have been conducted to determine the efficacy and toxicity of neoadjuvant chemotherapy (NAC) with irinotecan (CPT-11) and nedaplatin (NDP) followed by radical hysterectomy and adjuvant chemotherapy for locally advanced, bulky stage IB2-IIB cervical cancer [10–13]. Postoperative chemotherapy with CPT-11 and NDP without radiotherapy was also found to be very effective in high-risk patients with node-positive cervical cancer [14]. Abou-Taleb et al. reported that the CPT-11/NDP regimen shows favorable prognostic outcomes and lower toxicities than CCRT [15]. In our institute, chemotherapy has mainly been used for adjuvant treatment when complete resection of the cervical tumor is considered to have been achieved, even if high recurrence-risk factors are observed in postoperative pathological findings. We also administer chemotherapy using CPT-11 plus NDP for stage IB and II squamous cell carcinoma (SCC) of the uterine cervix.

In daily clinical practices, Uridine diphosphate glucuronosyltransferase 1 family polypeptide A1 (*UGT1A1*) genotyping is performed before treatment to estimate the degree of CPT-11 side-effects. UGT1A1 glucuronidates an active metabolite of irinotecan, SN-38. UGT1A1 protein glucuronidates SN-38 more than the other isoforms. Furthermore, *UGT1A1* genotypes affect the pharmacokinetics of SN-38 and its associated toxicity [16]. Patients with *UGT1A1* polymorphisms exhibit significantly higher response rates to NAC than those with wild-type *UGT1A1* (79.5% vs. 49.5%, *p* < 0.05), suggesting that *UGT1A1* may also serve as a highly potent marker for predicting the efficacy of NAC [17]. Therefore, we determined the influence of *UGT1A1* polymorphism on the prognosis, specifically progression-free survival (PFS), of local cervical cancer patients treated with CPT-11/NDP, including in patients at a high risk for recurrence. We also determined whether CPT-11/NDP was more effective as adjuvant chemotherapy in patients with *UGT1A1* polymorphism by further stratification of patient risk factors.

## Methods

### Patient registration

**Fig. 1:**
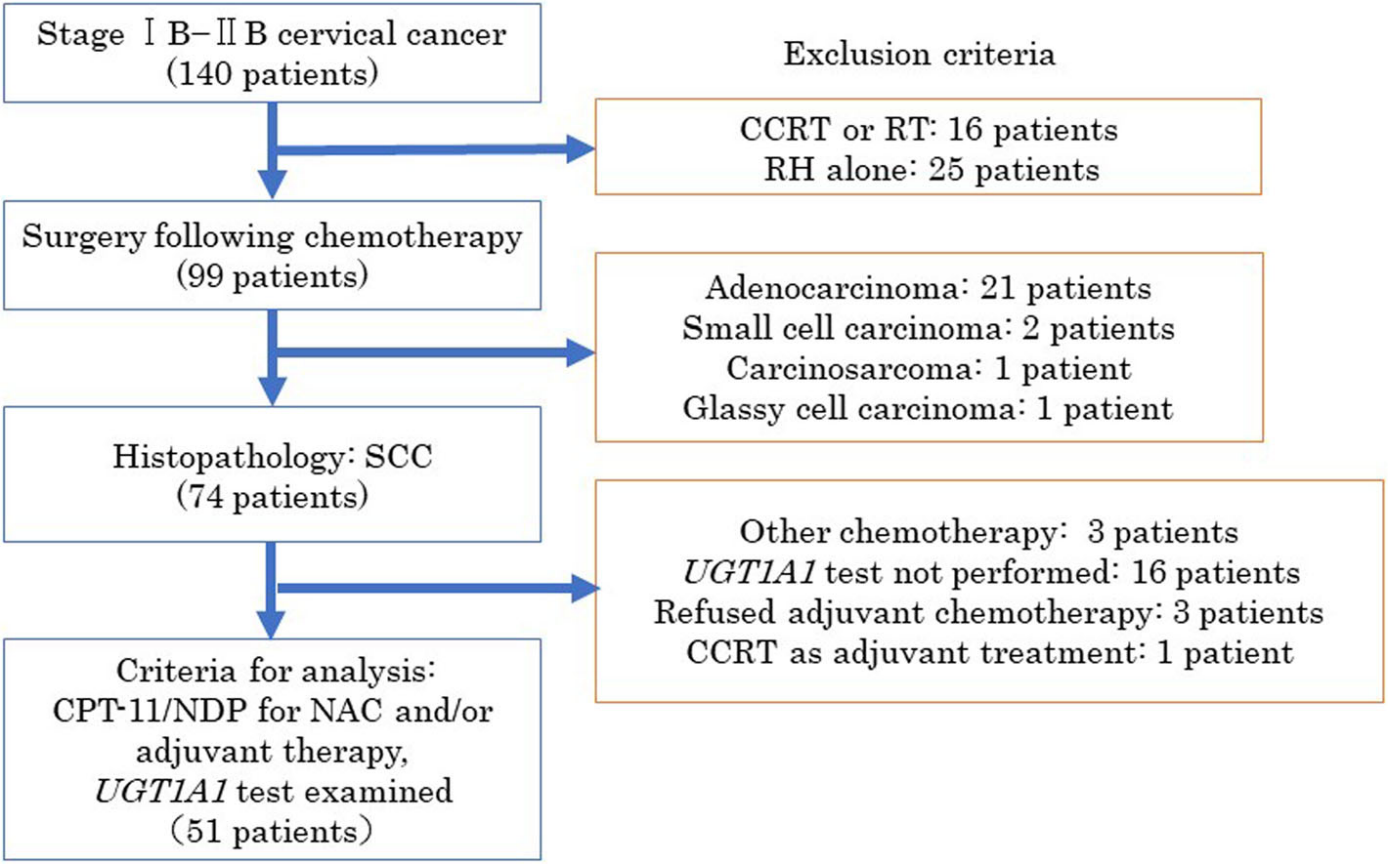
Patient selection process. CCRT: concurrent chemoradiotherapy; RT: radiotherapy; RH: radical hysterectomy; SCC: squamous cell carcinoma; CPT-11: irinotecan; NDP: nedaplatin; NAC: neoadjuvant chemotherapy; *UGT1A1*: uridine diphosphate glucuronosyltransferase 1A1

Figure 1 shows the patient selection process. In total, 140 patients with the 2009 FIGO stage IB-IIB uterine cervical cancer were treated at our hospital between 2010 and 2015. Forty-one patients treated with CCRT or surgery alone and 25 patients with histology other than SCC were excluded. We excluded three patients because they received chemotherapy other than CPT-11/NDP. We also excluded 16 patients without a *UGT1A1* test, 3 patients who refused adjuvant chemotherapy, and 1 patient who had positive margins in the resected tissue and was subsequently treated with CCRT as adjuvant treatment. The CPT-11/NDP regimen as neoadjuvant and/or adjuvant chemotherapy was used for all remaining patients (*n* = 51) due to patient risk factors. We performed further analyses on these 51 patients to examine the relationship between the effectiveness of CPT-11/NDP chemotherapy and *UGT1A1* genotype.

This retrospective study was approved by the ethics committee review board of Kyoto University Graduate School and Faculty of Medicine (approval number G531), and the requirement to obtain informed consent was waived because of the retrospective design; however, general written informed consent was obtained.

### Primary treatments

Clinical staging was performed by internal examination before the initial treatment. Lymph node metastasis was determined by a postoperative histopathological diagnosis of surgical specimens. All patients underwent radical hysterectomy and received systematic pelvic lymphadenectomy. Patients with stage IIB (*n* = 25), IIA2 (*n* = 1), IIA1 (*n* = 1), IB2 (*n* = 11), and IB1 (*n* = 13) disease with bulky tumors greater than 3.5 cm were also treated with neoadjuvant chemotherapy (*n* = 38, 74.5%). When intraoperative rapid diagnosis revealed pelvic lymph node metastasis, patients also received para-aortic lymphadenectomy.

The CPT-11/NDP regimen as NAC comprised of the intravenous administration of CPT-11 (60 mg/m^2^) on days 1 and 8 and NDP (80 mg/m^2^) on day 1 of a 21-day cycle, according to the JGOG1065 trial regimen [12]. Two patients received one cycle of NAC and 36 patients received two cycles of NAC. The CPT-11/NDP regimen as adjuvant chemotherapy comprised of the administration of CPT-11 (60 mg/m^2^) on days 1 and 15 and NDP (60 mg/m^2^) on day 1 of a 28-day cycle – a modified version of the regimen in the JGOG1067 trial comprising of the administration of CPT-11 (60 mg/m^2^) on days 1 and 8 [14]. A total of six cycles, including NAC and adjuvant chemotherapy, was considered a completion of the therapy. An average of 5.4 cycles of CPT-11/NDP were administered (six cycles, *n* = 35; five cycles, *n* = 8; four cycles, *n* = 5; three cycles, *n* = 1; two cycles, *n* = 1; and one cycle, *n* = 1). Only one patient received paclitaxel and carboplatin (four cycles) as adjuvant chemotherapy after 2 cycles of CPT-11/NDP as NAC due to a slight shrinkage ratio (20% decrease in tumor size).

*UGT1A1* genotypes were detected from patients’ blood. We categorized patients into two groups: wild-type (*1/*1) and polymorphism (*1/*6, *1/*28, *6/*6, or *28/*28). For patients with heterozygotic polymorphisms (*1/*6 or *1/*28), we did not reduce the dose of CPT-11. Of four patients with homozygotic (*6/*6 and *28/*28) or compound heterozygotic (*6/*28) polymorphisms, we reduced the dose of CPT-11 in only one patient (50 mg/m^2^) because she desired to avoid side effects. The other three patients received the normal CPT-11 dose and were closely monitored. Only in one patient the dose of NDP was reduced at the 2nd cycle of chemotherapy due to grade 3 nausea, and one patient experienced NDP allergic reactions; therefore, NDP was replaced with cisplatin from the second cycle in this patient. We assessed the side-effects using the Common Terminology Criteria for Adverse Events version 5.0 (https://ctep.cancer.gov/protocolDevelopment/electronic_applications/ctc.htm).

### Follow-up

All patients regularly underwent a physical examination, measurement of serum tumor markers, and imaging examinations, mainly computed tomography. Patients in this study were followed-up until May 2019. The median follow-up time was 60 months.

### Statistical analysis

We analyzed the relationship between PFS and clinical variables, including age, FIGO stage I versus II, *UGT1A1* genotype, and the number of metastatic lymph nodes. We used the R statistical software (version R-3.4.3, https://cran.ism.ac.jp/bin/macosx/, “The R Foundation for Statistical Computing,” Vienna, Austria). To identify the most important factors related to prognosis, conditional inference tree analysis was performed using the “party” package (https://cran.r-project.org/web/packages/party/index.html) with a univariate setting. Kaplan–Meier analyses and log-rank tests were performed using the “survival” package. We used Fisher’s exact test for the analysis of side effects. *P*-values < 0.05 were considered statistically significant.

## Results

### Background characteristics

The clinical backgrounds of all 51 patients are listed in Table 1.

**Table 1:** Clinical background: UGT1A1 genotype and clinical characteristics

*UGT1A1*		Total	*UGT1A1* wild-type	*UGT1A1* polymorphism (hetero/homo type)	*P*-value
Number (%)		51	24 (47.0%)	23/4 (45.1%/7.8%)	
Age, years	Average (min-max)	52.2	52.3 (36–64)	52.1 (29–78)	0.96
FIGO stage	IB1–2	24 (47.1%)	11	13	0.56
	IIA	2 (3.9%)	0	2	
	IIB	25 (49.0%)	13	12	
Lymph node metastasis	None	38 (74.5%)	17	21	0.44
	Pelvic nodes	11 (21.5%)	5	6	
	Para-aortic nodes	2 (3.9%)	2	0	

*FIGO* Federation of Gynecology and Obstetrics, *UGT1A1* Uridine diphosphate glucuronosyltransferase 1 family polypeptide A1

The mean patient age was 52.2 years, and there were 24 patients with stage IB disease (47.1%; IB1: *n* = 13 and IB2: *n* = 11), 2 patients with stage IIA disease (3.9%; IIA1: *n* = 1 and IIA2: *n* = 1), and 25 patients with stage IIB disease (49.0%). Twenty-four (47.1%) patients had wild-type *UGT1A1* (*UGT1A1* *1/*1), 23 (45.0%) patients had a heterozygotic polymorphism (*1/*6 or *1/*28), and 4 (7.8%) patients had a homozygotic (3 patients with *6/*6) or compound heterozygotic (1 patient with *6/*28) polymorphism. Pathological findings revealed pelvic node metastasis without para-aortic node metastasis in 11 (21.6%) patients and pelvic node metastasis with para-aortic node metastasis in 2 (3.9%) patients. Age, FIGO stage, and the number of metastatic lymph nodes were not different based on the *UGT1A1* genotype (Table 1).

### Tree-structured survival model

**Fig. 2:**
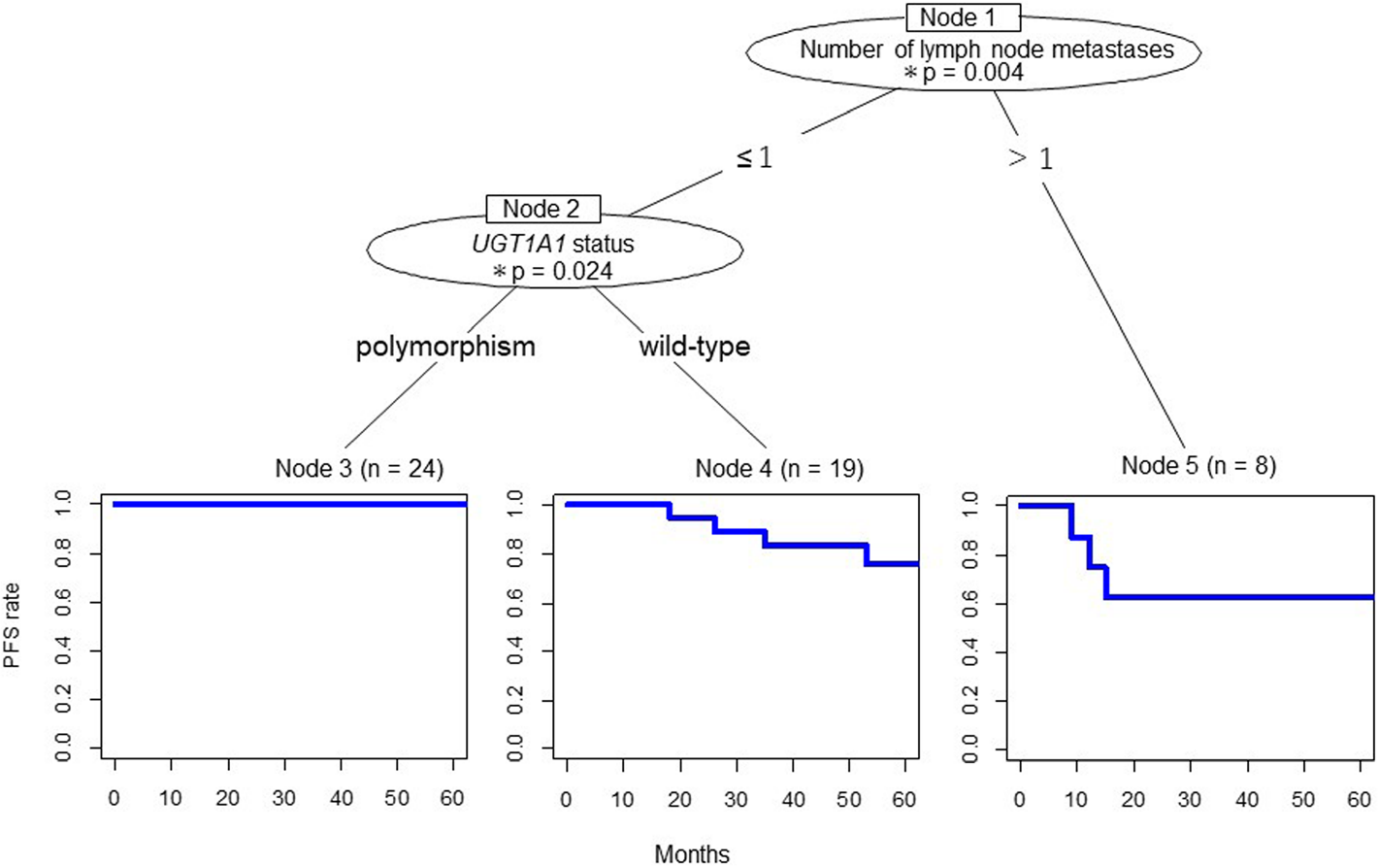
Tree-structured survival model. More than one metastatic lymph node was a primary determining prognostic factor (*p* = 0.004). *UGT1A1* polymorphism was a secondary determining high-risk factor for recurrence (*p* = 0.024). PFS: progression-free survival; meta: metastasis; *UTG1A1*: uridine diphosphate glucuronosyltransferase 1A1; *p* < 0.05*

We created a tree-structured survival model from our clinical variables including age, FIGO stage I versus II, UGT1A1 genotype, and the number of metastatic lymph nodes, to determine the most important factors related to PFS by univariate analysis. The primary determining prognostic factor for the risk of recurrence was two or more lymph node metastases upon pathological diagnosis (*p* = 0.004). The secondary stage of the tree-structured survival model showed that *UGT1A1* polymorphism was associated with a significantly better PFS than wild-type *UGT1A1* (*p* = 0.024) (Fig. 2). These findings suggest that a CPT-11/NDP regimen could be effective for patients with *UGT1A1* polymorphism and with one or no metastatic lymph nodes.

### The relationship between PFS and lymph node metastasis

**Fig. 3:**
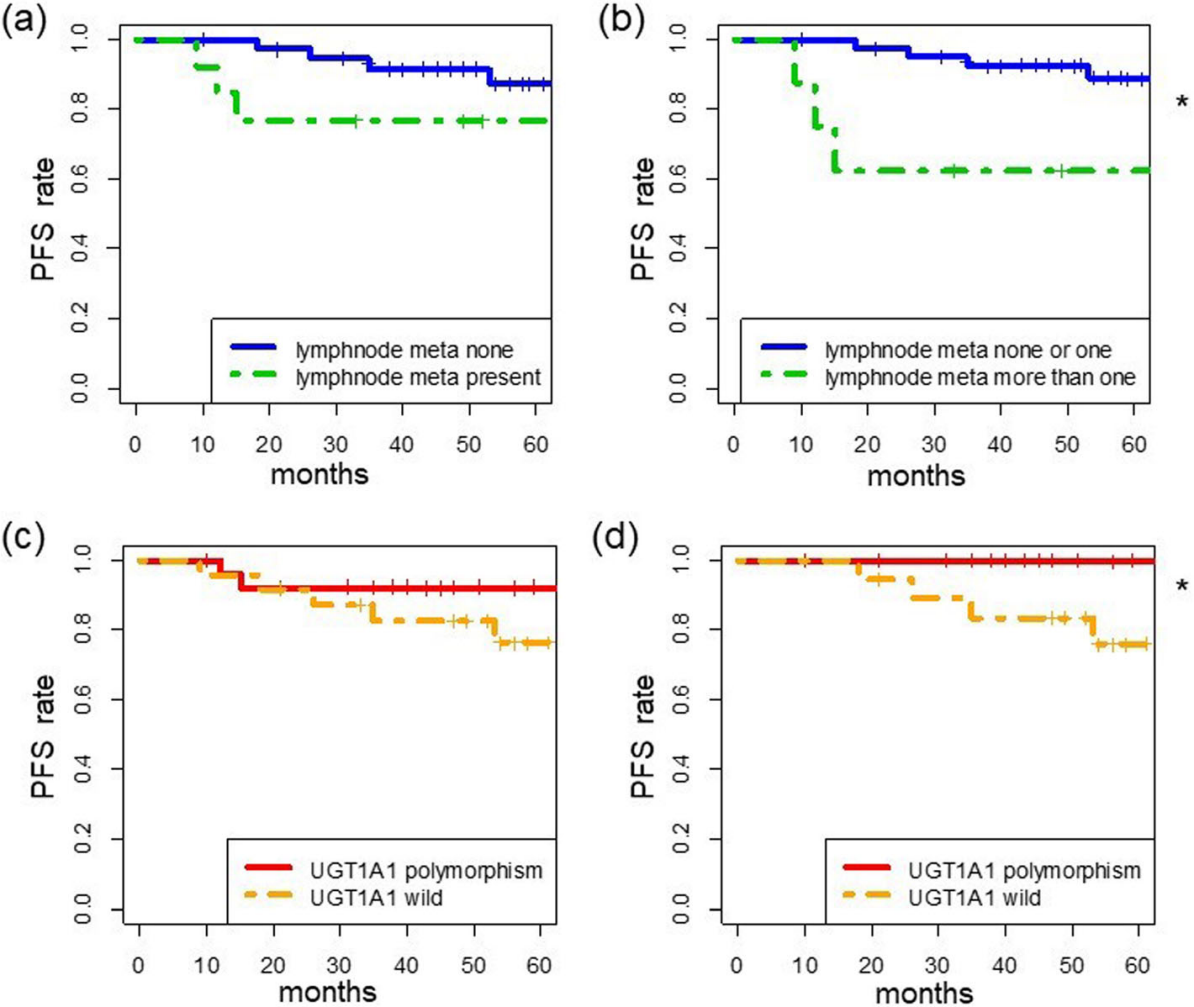
Progression-free survival (PFS) in cervical carcinoma patients. PFS based on **a** lymph node metastasis (*p* = 0.20), **b** number of lymph node metastases (*p* = 0.01), **c***UGT1A1* genotype (*p* = 0.20), and **d***UGT1A1* genotype in patients with ≤1 metastatic lymph node (*p* = 0.02). *UTG1A1*: uridine diphosphate glucuronosyltransferase 1A1; *p* < 0.05*

There was no significant difference in PFS between patients with and without lymph node metastasis (*p* = 0.20) (Fig. 3a). However, there was a tendency for better prognosis in patients without lymph node metastasis. Further, there was a significant difference in PFS between patients with none or one metastatic lymph node and those with more than one metastatic lymph node (*p* = 0.01) (Fig. 3b). Despite this limited analysis, we hypothesized that more than one metastatic lymph node might be a prognostic factor, as opposed to none or one metastatic lymph node.

### The relationship between PFS and *UGT1A1* genotype

There was no significant difference in PFS between patients with wild-type and polymorphic *UGT1A1* (*p* = 0.20) (Fig. 3c). However, there was a tendency for a better prognosis in patients with *UGT1A1* polymorphism. When we limited the analysis to patients with one or no metastatic lymph nodes, we found that patients with polymorphisms had a significantly longer PFS and no recurrence than patients without polymorphisms (*p* = 0.02) (Fig. 3d).

### Kaplan-Meier survival curves of overall survival (OS) and PFS among clinical stages

**Fig. 4:**
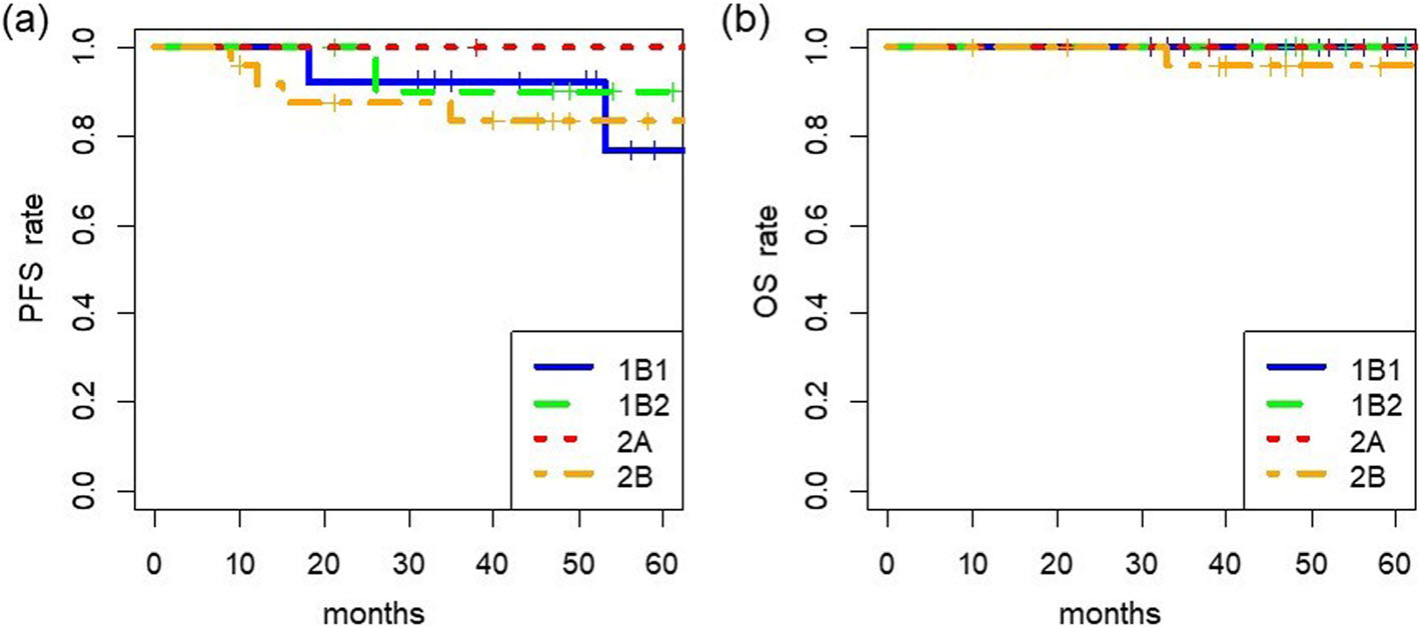
Survival in cervical cancer patients based on FIGO stage. **a** Progression-free survival (PFS) and **b** overall survival (OS)

The median PFS period was 55 months, and the median OS period was 60 months (5 years). The minimum follow-up period was 3 years and 6 months. The survival curves based on the FIGO stage are shown in Fig. 4. The 3.5-year PFS rates were 92% in stage IB1 patients, 90% in stage IB2 patients, 100% in stage IIA patients, and 83% in stage IIB patients (Fig. 4a). OS curves based on stage are shown in Fig. 4b. The 3.5-year OS rates were 100% in stage IB1 patients, 100% in stage IB2 patients, 100% in stage IIA patients, and 96% in stage IIB patients.

### Adverse events

We also analyzed the adverse events of chemotherapy. Grade 3 and 4 neutropenia occurred in 7 (29.1%) patients with wild-type *UGT1A1* and 15 (55.6%) patients with *UGT1A1* polymorphism. Neutropenia occurred more frequently, but not significantly, in patients with *UGT1A1* polymorphism than in patients without UGT1A1 polymorphism (*p* = 0.09). There was no significant difference in the incidence of other adverse events based on the *UGT1A1* genotype (Table 2). Additionally, there was no treatment-related death.

**Table 2:** Adverse events of CPT-11/NDP chemotherapy

*UGT1A1*	Wild-type (24 cases)	Polymorphism (hetero/homo, *n* = 23/4 cases)	*P*-value
Neutropenia	7	15	0.09
Grade 3	5	12	0.13
Grade 4	2	3	> 0.99
Febrile neutropenia	1	1	> 0.99
Nausea (> Grade 3)	1	2	> 0.99
Diarrhea (> Grade 3)	4	3	0.69
Anorexia (> Grade 3)	0	2	0.49
Thrombocytopenia (> Grade 3)	0	1	> 0.99

*CPT-11/NDP* Irinotecan/nedaplatin, *UGT1A1* Uridine diphosphate glucuronosyltransferase 1 family polypeptide A1

## Discussion

This tree-structured survival model implied that patients should be stratified first by the number of metastatic lymph nodes and second by *UGT1A1* genotype to help determine the risk of recurrence. We believe that it might be beneficial to administer CPT-11/NDP chemotherapy in patients with one or no lymph node metastases and *UGT1A1* polymorphism. A conditional inference tree is an effective way to determine and rank prognostic factors [18, 19].

We found that cervical cancer patients with one or no metastatic lymph nodes are less likely to experience recurrence after CPT-11/NDP therapy. It has been reported that the number of metastatic pelvic lymph nodes (≤3 vs. > 3) is a significant prognostic factor in patients treated with radical surgery followed by postoperative CCRT. Further, no significant survival difference is observed between patients without metastasis and those with 1–3 metastatic lymph nodes [20]. Park and Bae reported that the 5-year OS rates for patients with stage IB-IIA cervical cancer and 0, 1, and ≥ 2 positive metastatic lymph nodes were 91, 80, and 47%, respectively (*P* = 0.006) [21]. Inoue and Morita reported that the 5-year OS rates for patients with stage IB-IIB cervical cancer and 0, 1, 2–3, and ≥ 4 positive metastatic lymph nodes were 89, 81, 41, and 23%, respectively [22]. Sakuragi et al. reported that the cumulative 5-year OS rates for patients with 1 and ≥ 2 positive metastatic lymph nodes were 84.9 and 26.5%, respectively, with no significant difference between the cumulative OS rates of patients with 0 positive node and those with 1 positive node [23]. Therefore, ≥2 positive metastatic lymph nodes might be an important prognostic factor, rather than just an implicator of lymph node positivity.

Chemotherapy and surgery may be useful for patients with one or no lymph node metastasis. Nevertheless, we need to consider CCRT as adjuvant therapy, rather than chemotherapy alone, for patients with two or more lymph node metastases. We consider that the stratification of treatment based on the number of the lymph node metastases is preferable.

In patients with a history of radiation therapy, chemotherapy is the only course of treatment recommended when local recurrence is found in the vicinity of the pelvic cavity. We believe that secondary surgery or radiation therapy should be administered for local recurrence if patients have no history of radiation therapy [11]. Some studies advocated the use of chemotherapy or CCRT as initial adjuvant treatment after radical hysterectomy; however, the findings of such studies are inconclusive. We also believe that consolidation chemotherapy might lead to a better prognosis in patients with locally advanced cervical cancer if they were initially treated with CCRT [24].

This study implied that the *UGT1A1* polymorphism might also stratify patients and act as a predictive prognostic factor for the efficacy of CPT-11/NDP in cervical cancer patients. The *UGT1A1* genotype has previously been implicated as a prognostic marker for CPT-11 therapy in colorectal cancer cases [25]. Some controversial studies have suggested a limited survival benefit in patients who were UGT1A1-poor metabolizers due to *UGT1A1* polymorphisms [26, 27], although such an association has been inconsistently reported [28].

In our study, 43% of patients treated with chemotherapy experienced grade 3 or higher neutropenia, and 13.7% of patients experienced diarrhea and vomiting. Neutropenia and diarrhea are the common adverse effects of CPT-11. The *UGT1A1* genotype is known to be a useful predictor of adverse effects [29]. In our study, we categorized patients into the wild-type and polymorphism groups (*1/*6, *1/*28, *6/*6, *28/*28, and *6/*28), including a few patients with homozygotic or compound heterozygotic polymorphisms (5.9 and 2.0%, respectively). Patients with *UGT1A1* polymorphisms tended to experience grade 3 or 4 neutropenia more frequently than those with wild-type *UGT1A1* (*p* = 0.09, no significance). This finding is relatively consistent with reports showing that patients with *UGT1A1* homozygotic (*6/*6 or *28/*28) and compound heterozygotic (*6/*28) polymorphisms tend to experience adverse effects of CPT-11 [16, 30]. UGT1A1*28 and UGT1A1*6 have been well-studied *UGT1A1* polymorphisms in regards to CPT-11 pharmacokinetics and pharmacodynamics. Particularly in Caucasian patients, UGT1A1*28 seems to be a good predictor of neutropenia (at all CPT-11 doses) and diarrhea (at CPT-11 dose of 125 mg/m^2^). Additionally, UGT1A1*28 is also significantly associated with an increased risk of diarrhea in Asian patients at a CPT-11 dose of 125 mg/m^2^. However, in Asian populations, the UGT1A1*6 variant is more common and appears to be a more accurate predictor of neutropenia (all irinotecan doses) and diarrhea [31] than the UGT1A1*28 variant.

Our retrospective analysis revealed that there was a significant difference in PFS between the *UGT1A1* wild-type and polymorphism groups when we analyzed only patients with one or no lymph node metastases. Although we recommend CPT-11/NDP to patients with one or no lymph node metastases and *UGT1A1* polymorphism, our data do not support recommending this regimen to other patients. Nevertheless, we did not compare the efficacy and adverse effects of CPT-11/NDP to those of CCRT or other regimens, including paclitaxel/carboplatin or paclitaxel/cisplatin. Therefore, we should conduct a prospective study to test the more favorable prognostic effect of the CPT-11/NDP regimen in the *UGT1A1* polymorphism group than the wild-type group in cervical cancer patients with one or no lymph node metastases after radical hysterectomy.

## Conclusions

In conclusion, CPT-11/NDP might be beneficial in patients with cervical cancer, no or one metastatic lymph nodes, and *UGT1A1* polymorphism.

## Data Availability

Not applicable.

## Abbreviations

UGT1A1: Uridine diphosphate glucuronosyltransferase 1 family polypeptide A1
FIGO: International Federation of Gynecology and Obstetrics
HPV: Human papillomavirus
CCRT: Concurrent chemoradiotherapy
RT: Radiotherapy
RH: Radical hysterectomy
NAC: Neoadjuvant chemotherapy
CPT-11: Irinotecan
NDP: Nedaplatin
SCC: Squamous cell carcinoma
PFS: Progression-free survival
OS: Overall survival
